# Porcine Reproductive and Respiratory Syndrome (PRRSV2) Viral Diversity within a Farrow-to-Wean Farm Cohort Study

**DOI:** 10.3390/v15091837

**Published:** 2023-08-30

**Authors:** Mariana Kikuti, Carles Vilalta, Juan Sanhueza, Nakarin Pamornchainavakul, Jessica Kevill, My Yang, Igor A. D. Paploski, Tatiana Lenskaia, Nkechi M. Odogwu, Ross Kiehne, Kimberly VanderWaal, Declan Schroeder, Cesar A. Corzo

**Affiliations:** 1 Department of Veterinary Population Medicine, University of Minnesota, Saint Paul, MN 55108, USA; mkikuti@umn.edu (M.K.); carles.vilalta@irta.cat (C.V.); jsanhueza@uct.cl (J.S.); pamor001@umn.edu (N.P.); j.kevill@bangor.ac.uk (J.K.); ipaplosk@umn.edu (I.A.D.P.); lensk010@umn.edu (T.L.); odogw002@umn.edu (N.M.O.); kvw@umn.edu (K.V.); dcschroe@umn.edu (D.S.); 2 Unitat mixta d’Investigació IRTA-UAB en Sanitat Animal, Centre de Recerca en Sanitat Animal (CReSA), Campus de la Universitat Autònoma de Barcelona (UAB), 08193 Bellaterra, Spain; 3 Institut de Recerca i Tecnologia Agroalimentàries (IRTA), Programa de Sanitat Animal, Centre de Recerca en Sanitat Animal (CReSA), Campus de la Universitat Autònoma de Barcelona (UAB), 08193 Bellaterra, Spain; 4 Departamento de Ciencias Veterinarias y Salud Pública, Facultad de Recursos Naturales, Universidad Católica de Temuco, Temuco 02950, Chile; 5 Centre for Environmental Biotechnology, School of Natural Sciences, Bangor University, Bangor LL57 2UW, UK; 6 Swine Vet Center P.A., St. Peter, MN 56082, USA; rkiehne@swinevetcenter.com

**Keywords:** porcine reproductive and respiratory syndrome, genetic variation, whole-genome sequencing, molecular epidemiology

## Abstract

Describing PRRSV whole-genome viral diversity data over time within the host and within-farm is crucial for a better understanding of viral evolution and its implications. A cohort study was conducted at one naïve farrow-to-wean farm reporting a PRRSV outbreak. All piglets 3–5 days of age (DOA) born to mass-exposed sows through live virus inoculation with the recently introduced wild-type virus two weeks prior were sampled and followed up at 17–19 DOA. Samples from 127 piglets were individually tested for PRRSV by RT-PCR and 100 sequences were generated using Oxford Nanopore Technologies chemistry. Female piglets had significantly higher median Ct values than males (15.5 vs. 13.7, Kruskal–Wallis *p* < 0.001) at 3–5 DOA. A 52.8% mortality between sampling points was found, and the odds of dying by 17–19 DOA decreased with every one unit increase in Ct values at 3–5 DOA (OR = 0.76, 95% CI 0.61–0.94, *p* = 0.01). Although the within-pig percent nucleotide identity was overall high (99.7%) between 3–5 DOA and 17–19 DOA samples, ORFs 4 and 5a showed much lower identities (97.26% and 98.53%, respectively). When looking solely at ORF5, 62% of the sequences were identical to the 3–5 DOA consensus. Ten and eight regions showed increased nucleotide and amino acid genetic diversity, respectively, all found throughout ORFs 2a/2b, 4, 5a/5, 6, and 7.

## 1. Introduction

The porcine reproductive and respiratory syndrome virus (PRRSV) has been present in the United States (U.S.) pig population for at least three decades, with the first cases identified in the late 1980s and the first viral isolation in 1991 [1,2]. PRRSV2 (*Betaarterivirus suid 2*) is the PRRSV species most prevalent in the U.S., comprising over 95% of all PRRSV sequences generated through a monitoring project representing over 50% of the U.S. breeding herd [3]. It quickly became one of the most important diseases affecting pigs due to the associated economic losses to the swine industry worldwide. PRRSV is a positive-stranded RNA virus, and as such, its RNA polymerase lacks proofreading capabilities, which leads to a relatively high mutation rate and rapid evolution [4].

In order to understand disease dynamics, diagnostic tools/techniques to identify and characterize viral diversity have been rapidly evolving, from restriction fragment length polymorphism (RFLP) comparison to phylogenetic analysis and the classification of subtypes and clades using sequences targeting the open reading frames (ORFs) that encode structural proteins like ORF5 and ORF7 [5,6,7,8,9,10]. Briefly, RFLP-typing consists of interpreting the unique patterns generated through the fragmentation of a DNA or RNA sample by restriction enzymes. For PRRSV, RFLP-typing was initially proposed to differentiate wild (field) variants from vaccine-derived variants [5]. Even though RFLP-typing has been extensively used as an approach to classify PRRSV diversity, it still cannot accurately determine the genetic relatedness of PRRSVs, since single mutations at specific sites might classify highly homologous sequences into different RFLP patterns, making similarity interpretation problematic [11]. On the other hand, Sanger sequencing uses oligonucleotide primers to target specific regions of DNA or RNA. For PRRVS, targeting specific ORF regions also has limitations, such as the need for primers to be updated periodically since PRRSV mutates over time [4,7]. In addition, phylogenetic analysis of a single ORF may not fully represent the molecular diversity of the virus [12]. The error-prone nature of the virus, associated with its quick replication in the host, produces a mutational swarm of virions, also known as quasispecies [13,14]. Unfortunately, quasispecies are not captured through Sanger sequencing of PCR products, which has been the main tool used for genetic diversity studies in the past, since it generates a consensus sequence that likely represents the most prevalent viral sub-population present in a sample [4], in addition to being costly and time-consuming. The consensus sequence can still be informative; however, it can potentially limit the detection of multiple viruses present within a herd when pooled samples are sequenced during routine PRRSV monitoring. Instead, when pooling samples, the most prevalent virus represented in the pool’s consensus might not represent the most prevalent virus at the farm if the viral load of a single sample is significantly higher than the rest.

Due to PRRSV’s high evolutionary rate [15], it is unclear how viral diversity within a herd or an individual pig relates to vaccine efficacy, diagnostics, and immunopathogenesis, among others [4]. Further, PRRSV2 whole-genome data pinpointing viral diversity over time within the host or farm are still scarce, resulting in a knowledge gap that must be addressed to better understand viral evolution and its implications [13]. Although within-animal and within-farm differences accounted for around 3% of the ORF5 genetic variability when assessing quasispecies at two different farms at one sampling point [16], whole-genome variability over time within a naturally infected herd and within individuals is expected to be higher and more informative, but has yet to be documented. Moreover, changes in the PRRSV sequences over time may provide information on the general nature of mutations or substitutions that occur, which can lead to the production of escaping variants that might be a source of the next re-emergence of the virus. Thus, the main goal of this study was to characterize PRRSV2 diversity over time within infected piglets in a sow-farm that performed a live virus inoculation after an outbreak was detected.

## 2. Materials and Methods

A cohort study was conducted at one Midwestern U.S. 2500-sow farrow-to-wean farm, representative of current pig production practices, to characterize within-piglet diversity over time. The farm was considered PRRSV-naïve (i.e., status 4 according to the American Association of Swine Veterinarians [17], and expected to be ELISA negative) for over 2 years before this outbreak. The selected farm reported a PRRSV outbreak on 21 May 2019, with a virus classified as RFLP pattern 1-3-4 and lineage 1E. The veterinarian decided to reestablish the herd’s naïve status and proceeded to work towards virus elimination, with the first step being a mass viral exposure to generate herd immunity through live virus inoculation (LVI) on 30 May 2019. All sows and gilts were intramuscularly inoculated with the recently introduced virus obtained during the early stages of the outbreak from the serum of viremic on-site suckling piglets.

We aimed for a convenience sample size of 10 randomly selected litters from one farrowing room (representative of nearly 20% of the room). Considering that piglet viremia may persist for at least a month [18], 74% of the piglets from gilts experimentally exposed at 95 days of gestation are born viremic [19], and with an average of 11 born alive piglets per litter, we would expect 82 piglets to be PRRSV-positive at baseline. Additionally, assuming a 25% pre-weaning mortality during the outbreak [20] and an 84% PRRSV viral isolation positivity rate in piglets at weaning based on experimental infections during late-stage gestation [21], we would expect a follow-up of 82 piglets in which 69 were expected to be viremic. This sample size balanced budget constraints while considering the descriptive goals of this study. However, the PRRS outbreak heavily impacted this farm, forcing us instead to enroll all live piglets within the eligible age group available at the farm to obtain a similar sample size as the originally intended goal. On 12 June 2019, researchers visited the farm for the first time and collected blood from all suckling piglets 3–5 days of age (DOA). Sampled piglets were ear-tagged to ensure follow-up. The second blood sampling event occurred prior to weaning on 26 June 2019, when piglets were 17–19 DOA. These animals continued to be sampled over time through the nursery (at both 58 and 86 DOA) and finishing (at 127 DOA) phases.

Blood samples were centrifuged at 4 °C, 1500× *g*, for 10 min, and serum samples were collected, aliquoted, and stored at −80 °C until testing. Serum samples were individually tested for PRRSV by RT-PCR at the University of Minnesota Veterinary Diagnostic Laboratory. All positive samples from animals that completed follow-up were individually submitted for Oxford Nanopore Technologies (ONT) sequencing, as previously described [22]. Briefly, RNA was extracted using MagMAX-96 viral RNA isolation kit (Applied Biosystems, Thermo Fisher Scientific Inc., Waltham, MA, USA). Libraries were generated using the SMARTer universal low-input RNA kit for first-strand synthesis and prepared using the ligation sequencing kits. Libraries were individually barcoded and sequenced using the high-accuracy base-calling model with a minimum Q score of 7 using multiple FLO-MIN106 R9 flow cells on an ONT MinION or GridION sequencing platform. Reads were reference-assembled against a set of PRRSV genome sequences. Consensus whole-genome sequences were generated from reads for each sample by mapping to the reference MH651739 [23] using minimap2 version 2.2.0 and coding sequences (CDS)/ORFs annotations were transferred to each one of them.

Loss to follow-up due to death was described as percent mortality. PRRSV RT-PCR cycle threshold (Ct) values obtained from the RT-PCR, as a proxy of viral load, were described by the sampling point and compared by the piglet’s sex and sow parity using the Kruskal–Wallis equality-of-populations rank test. A univariate mixed-effects logistic regression using litter as a random effect was used to estimate the odds of dying as a function of the Ct value as a continuous variable at the initial sampling, sex, and sow parity to identify early factors potentially associated with pre-weaning mortality at a significance level of 0.05. Similarly, the same model was performed specifying a full factorial interaction term between sex and Ct values at the first sampling as a continuous variable; estimate margins of responses were plotted to illustrate the probability of death as a function of these two variables. Specification errors for this model were assessed through linktest. The linearity of Ct values with the log odds of dying was assessed using the Box–Tidwell model.

Consensus sequences were aligned against each other with MAFFT version 1.4.0 and pairwise nucleotide identities were calculated using Geneious Prime version 2020.2.5 default calculation. The minimum and maximum nucleotide identities observed were described within each sampling point, within each litter, and within single pigs over time. The percent nucleotide identity of each sequence to the overall sampling 1 (3–5 DOA) consensus was also calculated to describe the distribution of diversity within litters and sampling points. Because percent identity can be influenced by the size of the genome region being compared, samples that were only partially sequenced in any of the ORFs 2–7 were also excluded from further analysis.

The nucleotide diversity (Π) of ORFs 2–7 was calculated considering the map-to-reference assembly [24,25]. Briefly, we calculated the number of differences between all possible two-by-two sample comparisons. The sum of all differences within a moving window of 31 nucleotides was divided by the number of pairwise comparisons (sliding window of 31 nucleotides and one nucleotide step size) and assigned to the midpoint of the window. This number was then divided by the number of pairwise comparisons and then standardized to the sequence length by dividing it by the total length of the ORFs 2–7 sequences to obtain the nucleotide diversity of each 31-nucleotide sliding window in relation to the overall genome. An arbitrary threshold of ≥0.001 was defined to identify genomic regions with increased genetic variations, which represents the 90th percentile of the nucleotide diversity found per 31-nucleotide window. Similarly, the same methodology was applied to the translated sequences to estimate regions with increased amino acid diversity using a sliding window of 11 amino acids and one amino acid step size, as well as an arbitrary threshold of ≥0.028 representing the 90th percentile of the amino acid diversity found. All statistical analyses were done using Stata 15 [26]. All alignment, mapping, and pairwise nucleotide identities were performed in Geneious Prime version 2020.2.5 (https://www.geneious.com (accessed on 25 July 2023)) using default settings. Pairwise distances of all sequences compared to the sampling 1 (3–5 DOA) consensus were calculated in MEGAX: Molecular Evolutionary Genetics Analysis version 10.1.8 [27] using maximum composite likelihood, and the codon positions included were 1st + 2nd + 3rd + Noncoding. All ambiguous positions were removed for each sequence pair (pairwise deletion option).

This study was approved by the University of Minnesota Institutional Animal Care and Use Committee under the protocol ID: 1902-36777A.

## 3. Results

### 3.1. PRRSV RT-PCR Results

**Figure 1:**
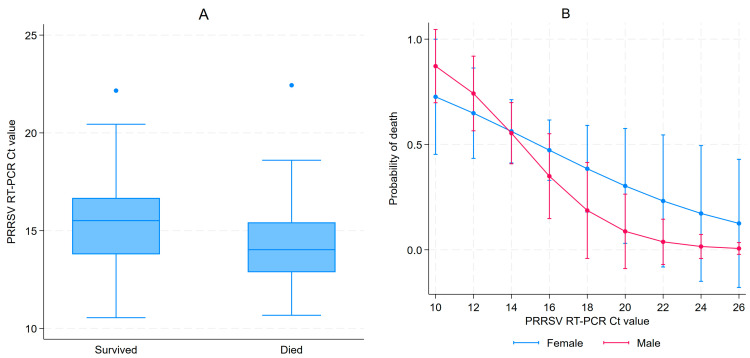
PRRSV Ct values at 3–5 days of age (DOA) distribution amongst piglets that survived or died before 17–19 DOA follow-up (**A**); and adjusted prediction of probability of dying according to sex and Ct values in 3–5 DOA (**B**).

A total of 127 piglets (67 females and 60 males) from 21 litters were enrolled in this study. These represented all live 3–5 DOA piglets available during the first farm visit. For most pigs, viremia persisted for up to 85 days of age, as illustrated by the cycle threshold (Ct) values shown in Figure S1 showing PRRSV Ct values at different ages, with negative RT-PCR results displayed as Ct 40. During the first visit (3–5 DOA), all samples tested RT-PCR positive for PRRSV with a median Ct value of 14.7 (IQR: 13.1–16.1). Females had significantly higher median Ct values at day 3–5 than males (15.5 (IQR: 13.8–16.5) vs. 13.7 (IQR: 12.8–15.3), Kruskal–Wallis *p* < 0.001). Ct values varied by parity at 3–5 DOA, as illustrated in Figure S2A. A 52.8% cumulative mortality between the first and second sampling event was observed, as 60 piglets had survived. Serum was collected from 59 of the 60 surviving piglets, since one piglet was in poor health and was spared from sampling. All piglets were still PRRSV positive with a median Ct value of 18.3 (IQR: 17.0–19.7). For the second sampling, RT-PCR Ct values for PRRSV did not vary by sex; the median for females was 18.2 compared to 18.5 for males (Kruskal–Wallis *p* > 0.999). Ct values by parity at 17–19 DOA are illustrated in Figure S2B. Ct values for most piglets increased between samplings, indicating a decrease in viremia, but decreased for seven pigs. Piglets that died between the 3–5 and 17–19 DOA samplings had significantly lower median Ct values at baseline than the surviving piglets (14.0 vs. 15.5, *p* = 0.001) (Figure 1A). After adjusting for litters, a 24% reduction in the probability of dying for each Ct incremental unit at 3–5 DOA (OR = 0.76, 95% CI 0.61–0.94, *p* = 0.01) was observed. The parity of the sow and the sex of the piglet were not associated with piglet mortality between the first and second sampling. The effect of sex and Ct values from the first sampling on the probability of dying is illustrated in Figure 1B.

### 3.2. ONT Sequencing Results

Sera from all 59 piglets that were sampled at both time points (i.e., 3–5 and 17–19 DOA) were used for ONT sequencing. We could not generate sufficient sequence coverage from the higher Ct-value samples throughout the remaining follow-up period (at 58, 86, and 127 DOA), thus ONT sequencing results represent solely the pre-weaning period of the 59 animals with paired samples (i.e., 118 samples). Although we obtained consensus sequences in ORFs 1a and 1b, the frequency of partial sequences was higher in ORF1 than in other ORFs. For this reason, we focused on the ORFs 2–7 region for analysis. Reads were obtained from 105 (54 from 3–5 DOA and 51 from 17–19 DOA) out of the 118 tested samples. An additional five sequences were excluded due to having a partial sequence in any of the ORFs 2–7 genes. These represented samples from five different animals originating from different litters, one collected at 3–5 DOA (female) while the remaining four were obtained at 17–19 DOA (one male and three females). Therefore, a total of 100 sequences were analyzed (53 from 3–5 DOA and 47 from 17–19 DOA) and are described henceforth. This represents paired samples from 43 piglets, 3–5 DOA-only samples from 10 piglets, and 17–19 DOA-only samples from four piglets. These piglets originated from a total of 20 litters, with a median of two piglets per litter (ranging from one to six piglets per litter). When assessing all ORFs 2–7 against each other (Table 1), the minimum percent nucleotide identity observed was 99.84% and 99.57% when comparing all samples collected at 3–5 and at 17–19 DOA, respectively. The minimum pairwise percent nucleotide identity per litter was calculated, and the minimum observed in any litter was 99.88% at 3–5 DOA and 99.57% at 17–19 DOA. The minimum within-pig percent identity between the first and second sampling was 99.65%. When assessing each of the ORFs separately, the percent identity amongst samples was overall high for most comparisons (>99.0%). The lowest overall per sampling point and within-litter percent nucleotide identity was 95.81%, found in ORF4 at 17–19 DOA. The second lowest percent nucleotide identity was the overall per sampling point 97.74% identity in ORF5a at sampling 1 (3–5 DOA), followed by 98.53% for the overall per sampling point and within-litter in ORF5a at sampling 2 (17–19 DOA). Similarly, the lowest percent nucleotide identity within an animal (across the first and second time point) was also found in ORFs 4 and 5a at 97.26% and 98.53%, respectively.

**Figure 2:**
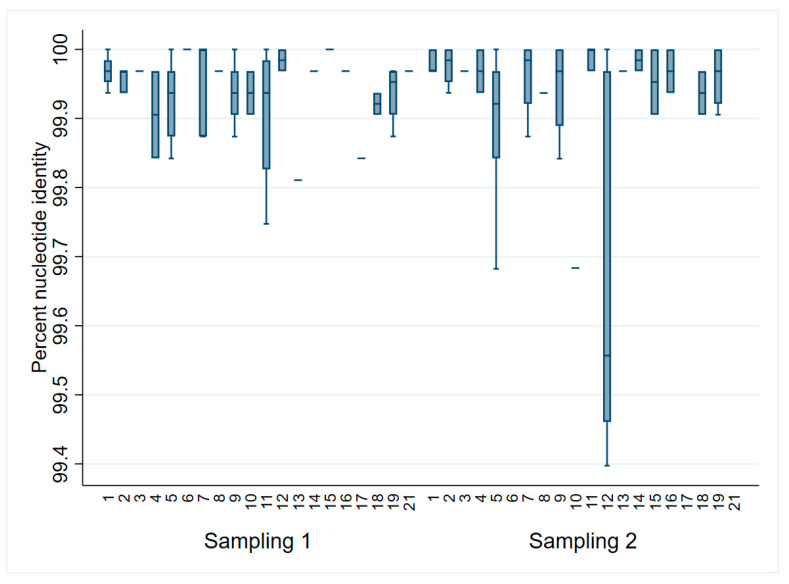
PRRSV ORFs 2–7 percent identity to the sampling 1 (3–5 days of age) consensus by litter and sampling point.

When comparing all sequences to the ORFs 2–7 sampling 1 consensus, sequences differed less than 1% from the consensus (Figure 2). However, only 31% (31/100) of all the sequences were identical to the consensus. Of those, 14 were from sampling 1 (3–5 DOA) and 17 were from sampling 2 (17–19 DOA). The most distant sequences to the consensus (<99.7% identity) were identified in litters 5, 10, and 12 during sampling at 17–19 DOA. When assessing solely ORF5, 62% (62/100) were identical to the sampling 1 consensus (33 from sampling 1 and 29 from sampling 2).

**Figure 3:**
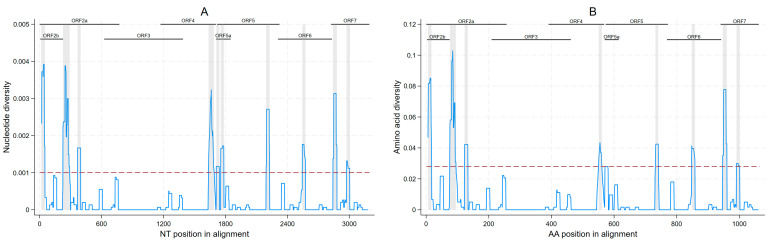
Within-farm nucleotide (**A**); and amino acid diversity (**B**) of porcine reproductive and respiratory syndrome within a 31-nucleotide or 11-amino-acid sliding window with increased diversity regions highlighted in grey (nucleotide diversity ≥0.001 or amino acid diversity ≥0.028 threshold).

Considering all ORFs 2–7 sequences generated, we observed areas of high and low nucleotide diversity throughout the genome, with 10 regions displaying an increased genetic variation throughout ORFs 2a/2b, 4, 5a/5, 6, and 7 (Figure 3A). These regions had a median of 31.5 nucleotides (minimum 30, maximum 65). Eight amino acid regions with increased diversity were found in ORFs 2a, 2b, 4, 5, 6, and 7 (Figure 3B), ranging from 9–19 amino acids in length (median of 10 amino acids). Sites in which any nucleotide differences were found are shown in Figure S3.

## 4. Discussion

A 53% pre-weaning mortality was recorded among the population of animals initially enrolled in the project. This is on the high end of the 25–50% pre-weaning mortality during an outbreak previously described in the literature [20]. Our data also found that Ct values, as a proxy for viral load, were lower among males than among females at baseline. This same finding was previously reported in an observational study with this age group [28]. Since samples were collected from 3–5 DOA piglets, it is unclear whether males are born with a higher virus load than females or if behavioral, biological, or physiological aspects established during the first days of life might help to explain that effect, or if this represents a confounding not accounted for in the univariate analysis. However, there was no difference in Ct values or in the mortality rate by sex at 17–19 DOA. This potentially suggests a complex relationship between sex and viremia, and that these differences found earlier in life might be diluted over time by constant exposure to the virions being shed by this pig population. Viremia persisted through weaning for all animals, as expected, since it has been shown that the duration of viremia is dependent on an animal’s age, being higher and longer in piglets compared to adults or grower–finishers [18,29]. Unfortunately, we were not able to obtain enough ORFs 2–7 sequences to be able to describe diversity in the grow–finishing phase.

Although PRRSV sequences from this piglet population were found to have high genetic similarity, a lower percent nucleotide identity was found in ORFs 4 and 5a. Both of these regions were previously described as regions with higher variability within the PRRSV-1 genome [30]. Additionally, ORF5a overlaps almost completely with the beginning of ORF5, where sites under positive selective pressure were previously described [9]. However, the percent nucleotide identity should be interpreted with caution since region length can potentially inflate the percent nucleotide differences (e.g., the impact of one mutation in the percent identity is higher when the segment length is shorter). The sites within ORF5 that were reported to be under positive selection [9] were not found to be among the ORF5 region with greater nucleotide diversity in this study. This difference can be potentially explained by the fact that studies reporting sites under selective pressure usually use sequence libraries comprised of either publicly available data or clinical samples (e.g., from veterinary diagnostic laboratories). Here, we systematically sampled all animals from a farm with an ongoing PRRSV outbreak, and those were followed through weaning age. While the former might be better at capturing the selected mutations that might represent a broader range of PRRSV-affected farms, the latter might be better at capturing random mutations that have not been cleaned out of the population yet. Time-clustered sampling has been suggested to more closely represent the mutation rate than the substitution rate [31]. However, both are important for a full understanding of evolutionary dynamics [32]. Additionally, the only ORF5 region in which diversity was found to be higher than the threshold (representing the 90th percentile of the overall amino acid diversity in ORFs 2–7) was between amino acid positions 162–171, which are contained in regions potentially associated with T-cell and B-cell epitopes [33,34].

We observed that a lower percent nucleotide identity to the sampling 1 consensus occurred within the 17–19 DOA sampling, particularly in litters 5, 10, and 12. This highlights an increased genetic diversity over time, particularly considering that the PRRSV mutation rate at the ORFs 3–5 regions was estimated at the order of 10^−2^ substitutions/site/year [35]. Moreover, we found that the within-animal percent identity between samplings at 3–5 DOA and 17–19 DOA was as low as 99.65% for ORFs 2–7 and 97.26% for ORF4, indicating that up to 11 and 14 mutations were detected in these regions within 12 to 16 days, respectively. A possible explanatory hypothesis for this increased diversity after a follow-up of two weeks is that we might be observing the turnover of a co-circulating virus that was not previously detected during the first sampling, either at the farm level or at the within-animal quasispecies level. However, we need to consider the limitation that these differences were observed among the consensus sequences obtained from each animal at each sampling point and that the within-animal viral subpopulation was not assessed. Or, alternatively, some animals might have been initially infected by an already more diverse population of virions. What caused these differences to occur within particular litters remains to be elucidated. The co-circulation of multiple PRRSV variants within farms with different levels of PRRS historical exposure was reported based on ORF5 sequencing [36], and this highlights the complex nature of within-farm PRRS viral population dynamics. Increased within-farm viral diversity might be attributed to the constant exposure in gilts to either wild or vaccine viruses, to the new introduction of new field-PRRSV variants through biosecurity breaches, or to within-farm viral evolution. Similar to what was found when analyzing three isolates from nursery barns from a Lineage 1 PRRSV2-affected pig farm in China [37], the high similarity between sequences generated in our study suggests that diversity represents different evolutionary processes resulting from even a single introduction. However, we aimed to describe individual-level diversity over time, thus the same animals were sampled instead of randomly selecting animals at each sampling point. Because the response to infection might vary by individual, these results might not represent animals born to subsequent weekly batches. In fact, this could be illustrated by a recent study in which higher PRRSV1 diversity was found within batches 1.5 months after the outbreak onset than within batches 12 months after, possibly representing the emergence of an escape variant [38].

Another point that deserves attention is that 31% and 62% of all sequences were identical to the ORFs 2–7 and ORF5 overall consensus, respectively. This is important because, typically, producers and veterinarians submit samples from only one animal or, alternatively, from a pool of samples to be sequenced, and that one consensus sequence will be used for molecular epidemiology investigations. Thus, it is important to understand the limitations of such techniques, which might not fully represent the viral population co-circulating in a farm. Although this strategy might not allow for a comprehensive understanding of within-farm viral diversity, it might still be sufficient to guide outbreak control interventions. Consequently, the decision regarding a sampling and testing strategy should rely on the question that producers, veterinarians, or scientists aim to address, given the considerable costs associated with personnel time and sequencing.

Important limitations of this study are that animals were born to sows inoculated with the same wild-type virus found on the farm, meaning the within-farm PRRSV genetic diversity during the outbreak might have been artificially reduced due to the sows having all been exposed to the same virus. Thus, the genetic diversity within this highly homogeneous group of animals might not represent PRRSV genetic diversity assessed across the pig populations in which this control strategy was not implemented, or in which the sows had prior immunity before the outbreak. However, this control strategy is commonly used in the field, and is therefore partially representative of current practices. We also described diversity solely between animals in which the pre-weaning follow-up was complete due to budget constraints. The viral population in animals that died between 3–5 DOA and 17–19 DOA samplings could have shown different characteristics to those described amongst surviving piglets. However, because of how highly homogeneous PRRSV was found to be in general, it is likely that these differences were small. Additionally, both the nucleotide and amino acid regions with increased diversity were identified through an arbitrary threshold. Although no standardized threshold has been established for PRRSV nucleotide diversity, this method allows for the identification of areas with higher diversity within highly similar sequences and has also been used in other fields, such as SARS-CoV-2, with arbitrary thresholds [25]. Here, we attempted to add meaning to the threshold by using the 90th percentile to separate regions with nucleotide or amino acid diversity higher than 90% of all the regions in the ORFs 2–7 genome. The number and size of the regions with increased diversity might have been higher if a lower threshold had been applied.

## 5. Conclusions

Although the studied animals were born to sows that underwent live virus inoculation with the same inoculum yielding very closely related sequences, we still identified regions with increased nucleotide and amino acid diversity throughout different regions of the genome, including ORF4, in a short window of time. The ORF4 also showed the lowest minimum percent nucleotide identity throughout the course of two weeks. These findings highlight that, even in a scenario with expected low diversity, more intense sampling might be needed to truly characterize the viral population during an ongoing outbreak.

## Figures and Tables

**Table 1:** Overall per sampling point within-litter and within-pig PRRSV maximum and minimum pairwise percent nucleotide identity at 3–5 (sampling 1) and at 17–19 (sampling 2) days of age.

Percent Identity		Sampling 1 (S1)		Sampling 2 (S2)		Within Animals (S1 × S2)
		Overall	Within Litter	Overall	Within Litter	
ORF2–7	Minimum	99.84	99.88	99.57	99.57	99.65
	Maximum	100.00	100.00	100.00	100.00	100.00
ORF2a	Minimum	99.21	99.34	98.94	99.07	99.21
	Maximum	100.00	100.00	100.00	100.00	100.00
ORF2b	Minimum	99.07	99.07	98.60	99.06	99.06
	Maximum	100.00	100.00	100.00	100.00	100.00
ORF3	Minimum	99.74	99.87	99.74	99.74	99.87
	Maximum	100.00	100.00	100.00	100.00	100.00
ORF4	Minimum	99.63	99.81	95.81	95.81	97.26
	Maximum	100.00	100.00	100.00	100.00	100.00
ORF5a	Minimum	97.74	98.53	98.53	98.53	98.53
	Maximum	100.00	100.00	100.00	100.00	100.00
ORF5	Minimum	99.50	99.50	99.66	99.67	99.66
	Maximum	100.00	100.00	100.00	100.00	100.00
ORF6	Minimum	99.62	99.81	99.61	99.80	99.80
	Maximum	100.00	100.00	100.00	100.00	100.00
ORF7	Minimum	99.46	99.73	99.73	99.73	100.00
	Maximum	100.00	100.00	100.00	100.00	100.00

## Data Availability

The overall consensus for all sequences generated by this study have been deposited in GenBank under the accession number MZ423536, while individual reads are available under BioProject accession number PRJNA738550. Metadata associated with each sample are available upon request.

## Supplementary Materials

The following supporting information can be downloaded at: https://www.mdpi.com/article/10.3390/v15091837/s1, Figure S1: porcine reproductive and respiratory syndrome virus (PRRSV) cycle threshold (Ct) value of piglets born during an outbreak over time. Negative samples are presented as samples with Ct values of 40; Figure S2: PRRSV RT-PCR Ct value distribution of samples collected from piglets at 3–5 (A); and 17–19 (B) days old according to sow’s parity in a farrow-to-wean herd undergoing an outbreak; Figure S3: percentage of total observations each nucleotide was found in, in a given position. Positions in which no differences were found were suppressed.
