# Supplementary figures and images for: Porcine Reproductive and Respiratory Syndrome (PRRSV2) Viral Diversity within a Farrow-to-Wean Farm Cohort Study
Source: Viruses. 2023 Aug 30;15(9):1837. doi: 10.3390/v15091837 (PMC10535563; doi:10.3390/v15091837)

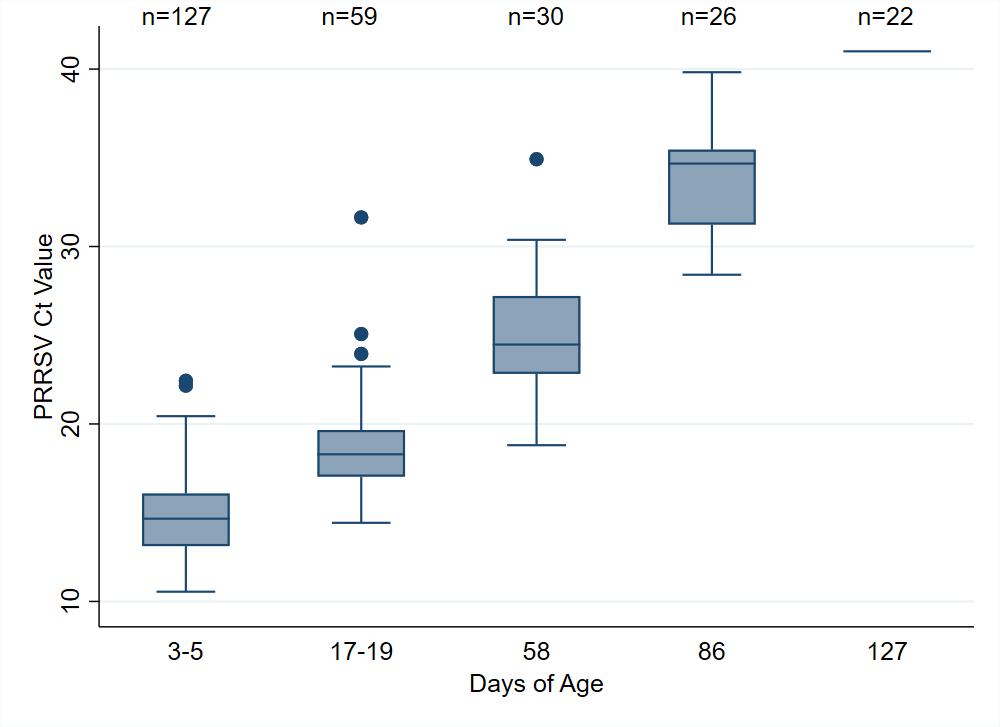

Supplement: Supplementary file 1 [file viruses-15-01837-s001.zip › viruses-2543467-supplementary-Figure S1.png]

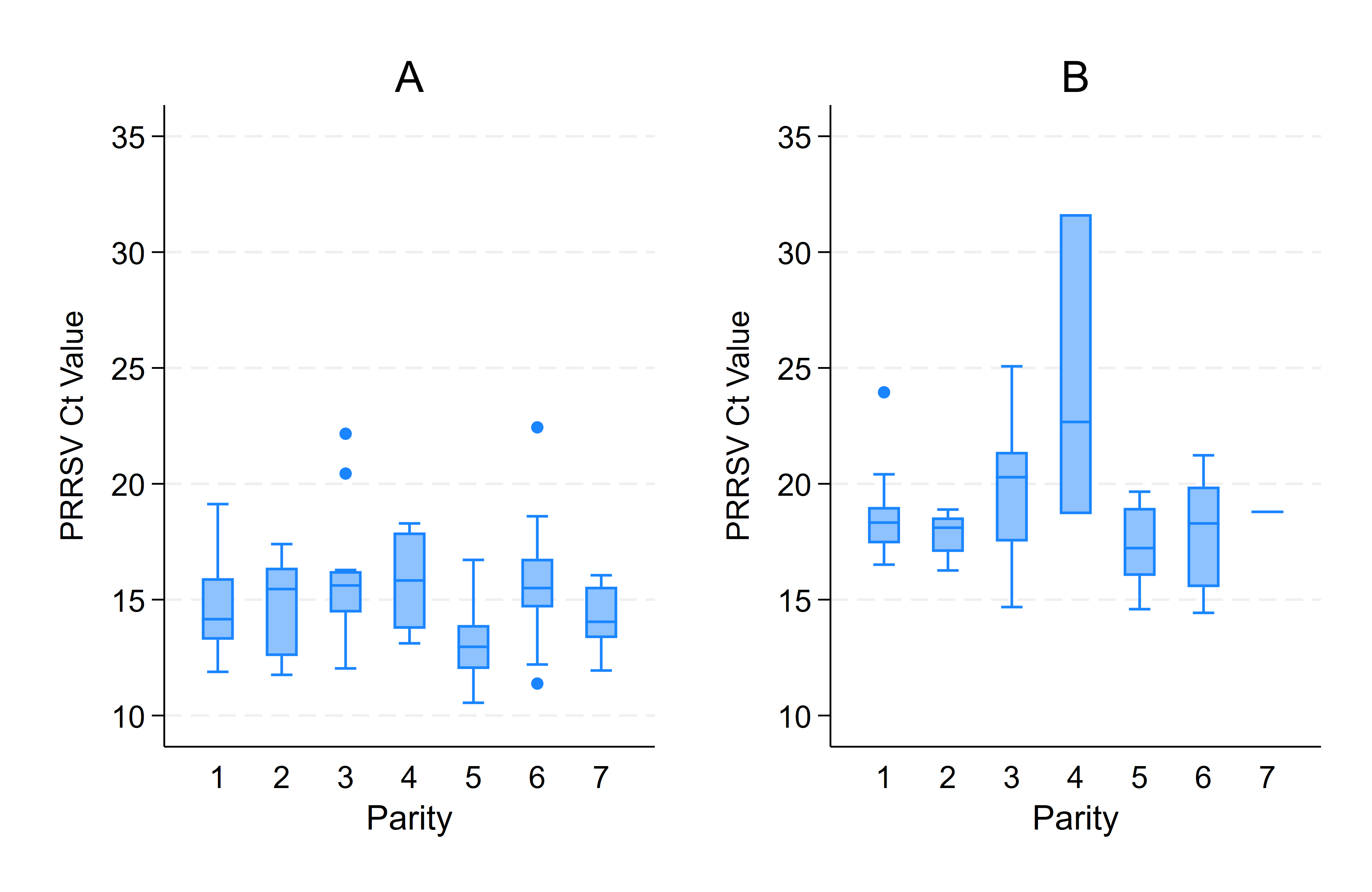

Supplement: Supplementary file 1 [file viruses-15-01837-s001.zip › viruses-2543467-supplementary-Figure S2.png]

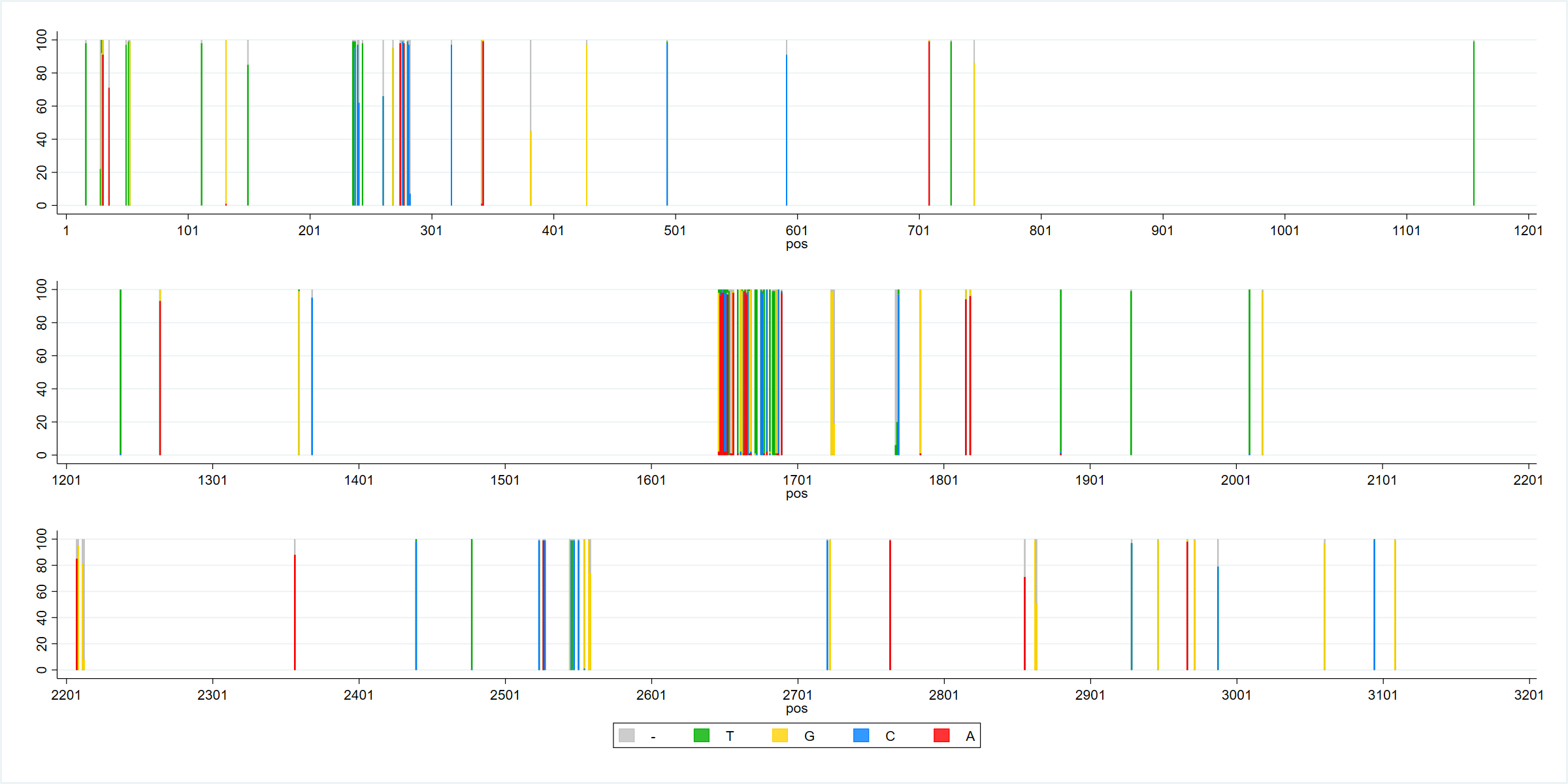

Supplement: Supplementary file 1 [file viruses-15-01837-s001.zip › viruses-2543467-supplementary-Figure S3.tif]
